# Cell type-specific functions of Alzheimer's disease endocytic risk genes

**DOI:** 10.1098/rstb.2022.0378

**Published:** 2024-04-08

**Authors:** Johanna-Katharina Maninger, Karolina Nowak, Srilakshmi Goberdhan, Rachel O'Donoghue, Natalie Connor-Robson

**Affiliations:** Cardiff University, Dementia Research Institute, Cardiff University¸ Cardiff, CF24 4HQ, UK

**Keywords:** endocytosis, endosome, *BIN1*, *PICALM*, *CD2AP*, *SORL1*

## Abstract

Endocytosis is a key cellular pathway required for the internalization of cellular nutrients, lipids and receptor-bound cargoes. It is also critical for the recycling of cellular components, cellular trafficking and membrane dynamics. The endocytic pathway has been consistently implicated in Alzheimer's disease (AD) through repeated genome-wide association studies and the existence of rare coding mutations in endocytic genes. *BIN1* and *PICALM* are two of the most significant late-onset AD risk genes after *APOE* and are both key to clathrin-mediated endocytic biology. Pathological studies also demonstrate that endocytic dysfunction is an early characteristic of late-onset AD, being seen in the prodromal phase of the disease. Different cell types of the brain have specific requirements of the endocytic pathway. Neurons require efficient recycling of synaptic vesicles and microglia use the specialized form of endocytosis—phagocytosis—for their normal function. Therefore, disease-associated changes in endocytic genes will have varied impacts across different cell types, which remains to be fully explored. Given the genetic and pathological evidence for endocytic dysfunction in AD, understanding how such changes and the related cell type-specific vulnerabilities impact normal cellular function and contribute to disease is vital and could present novel therapeutic opportunities.

This article is part of a discussion meeting issue ‘Understanding the endo-lysosomal network in neurodegeneration’.

## 1. Introduction

Alzheimer's disease (AD) is the most common neurodegenerative disease affecting 55 million worldwide, with the prevalence expected to more than double by 2050 [1]. Patients present with progressive memory impairment, confusion, disorientation, deterioration of speech and language and the progressive loss of executive functions making it increasingly difficult for them to carry out daily living independently. Familial AD is rare accounting for less than 5% of cases and it is caused by inheritance of mutations in the presenilin 1 (*PSEN1*), presenilin 2 (*PSEN2*) or amyloid precursor protein (*APP*) genes [2]. However, the remaining more than 95% of AD cases are classed as sporadic but it is still considered that 58–79% of the risk of developing the sporadic disease is genetic [3]. The genetic landscape of the sporadic disease is complex and points to a variety of cellular pathways being involved in its pathogenesis. These pathways include cholesterol and lipid metabolism, immune response and amyloid beta (Aβ) processing, which are reviewed in detail elsewhere [4–7]. This review will concentrate on the role of the endocytic pathway in the development of AD. Understanding the molecular mechanisms underlying this sporadic late-onset AD (LOAD) genetic risk represents a gap in our knowledge and is a bottleneck in producing effective therapies.

Genome-wide association studies (GWAS) have now identified over seventy risk loci associated with LOAD [8–12]. These studies have repeatedly demonstrated a cluster of genes within the endocytic pathway, including *BIN1*, *PICALM*, *CD2AP* and *SORL1*, among others*.* Single-nucleotide polymorphisms (SNPs) in these genes are associated with clinical phenotypes and rare LOAD-associated coding variants, further emphasizing their importance [13–15]. In addition to genetic studies, pathological evidence also highlights endocytic and endosomal dysfunction as key disease mechanisms, with early endosomal enlargement being observed as one of the earliest intraneuronal pathologies in LOAD and even being evidenced in the prodromal phase of disease [16]. Further to this, the neuronal early endosome (EE) is considered responsible for the primary production and secretion of amyloid beta 42 (Aβ42) through its role in APP processing [17]. Importantly, targeting the disruption seen in the endosomal pathway in LOAD through preclinical trials indicates its ability to ameliorate fundamental AD pathologies such as Aβ and tau accumulation and synaptic dysfunction [18–20].

The endocytic pathway is used to internalize extracellular material such as lipids and nutrients as well as plasma membrane- and receptor-bound material. These cargos are then trafficked through the endosomal pathway, a set of membrane bound organelles where it is sorted to its required destination. These pathways are interlinked and essential for the internalizing, sorting and recycling of cellular components. The endosomal pathway comprises the EE, recycling endosome (RE) and late endosome (LE), which together enable the sorting and recycling of components either back to the cell membrane or through the LE to the lysosome for degradation. Therefore, the pathway is also important in membrane composition and protein turnover. Across the brain different cell types are heavily reliant on functioning endocytosis and endosomal trafficking. Neurons and microglia are two important cell types in LOAD and in addition to the normal requirements of endocytic function described above, they have specific uses of the endocytic system. Neurons are post-mitotic, complex cells with extensive neuritic arbours to maintain, which makes correct cellular transport and recycling crucial. In addition, neurons are constantly releasing and recycling synaptic vesicles, which requires functional clathrin-mediated endocytosis (CME) [21]. Microglia have a central role in the surveillance and preservation of their neuronal surroundings and are important in maintaining synaptic health. They rely on endocytosis and endosomal function for antigen presentation and motility, and they use the specialized form of endocytosis—phagocytosis—to maintain a healthy brain environment [22,23]. Unlike CME, which internalizes cargos of less than 120 nm, phagocytosis is used to clear larger particles of greater than 500 nm, including pathogenic protein aggregates such as Aβ plaques, dead neurons and myelin debris [24]. Therefore, endocytic/endosomal dysfunction is likely to impact different cell types in specific manners, making it important to understand how this wider impact on cellular function can drive LOAD mechanisms.

**Figure 1.**
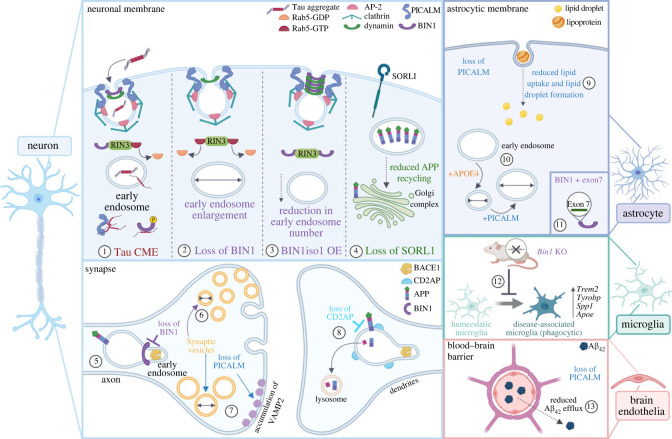
Pathways to endocytic dysfunction through LOAD endocytic risk genes. The endocytic pathway is required for the internalization of cellular substrates, receptor-bound cargos and nutrients, but it also has cell type-specific functions. Here we highlight roles of key LOAD-associated endocytic proteins. (1) CME acts as a route of Tau internalization in neurons. (2) Loss of BIN1 in neurons induces early endosome enlargement. Increased binding of RAB5 and RIN3 has been suggested as a possible cause. (3) Overexpression (OE) of the human BIN1iso1 in neurons reduces early endosome number. (4) Loss of SORL1 in neurons leads to reduced APP recycling, including retention of APP inside endosomes and reduction of APP in the Golgi. (5) Loss of BIN1 in neurons disrupts BACE1 recycling in axons. (6) Neurons of *BIN1* KO mice harbour increased reserve pools of presynaptic vesicles of reduced size, and more docked vesicles with decreased release probability. (7) Loss of PICALM in neurons leads to reduction in the number of presynaptic vesicles and vesicle clusters, enlargement of individual vesicles and cell surface accumulation of the synaptic protein VAMP2. (8) Loss of CD2AP in neurons prevents sorting of APP for degradation in dendrites. (9) Loss of PICALM in astrocytes leads to decreased lipid uptake from neurons and decreased lipid droplet formation. (10) Overexpression of PICALM in astrocytes was sufficient to begin to rescue the APOE4-induced early endosome size reduction. (11) Astrocytes express isoforms of BIN1 containing exon 7, which is associated with both Tau and amyloid pathology and AD-related cognitive decline. (12) Microglia of *BIN1* KO mice exhibit a dampened inflammatory response and downregulation of genes related to the disease-associated microglia (DAM) phenotype. (13) Loss of PICALM in brain endothelial cells leads to reduced efflux of Aβ_42_ from the brain across the blood-brain barrier.

Together, the genetic and pathological evidence linking the endocytic pathway and endosomal trafficking to LOAD and its potential as a novel therapeutic strategy make understanding the function of endocytic risk genes and their impact on different cell types crucial. Throughout this review, we aim to consider the current evidence for cell type-specific roles of LOAD endocytic risk genes, including in neurons, microglia, astrocytes and brain endothelia. To provide the reader with an overview of the main points in the review, we have summarized the main findings in figure 1 and electronic supplementary material, table S1.

## 2. PICALM

Multiple GWAS studies have identified and replicated *PICALM* as one of the most significant LOAD risk genes after *APOE* and *BIN1* [8–11]. SNPs in *PICALM* are found in non-coding regions and are thought to regulate PICALM expression. Post-mortem and expression quantitative trait loci (eQTL) studies support this, with PICALM expression being reduced in LOAD brains whereas protective *PICALM* SNPs are considered to increase its expression [25,26]. However, the impact of SNPs on different isoforms is likely more nuanced and further work is required in particular to understand how this impacts cell type-specific expression patterns [6,27]. For example, *PICALM* SNPs have been linked to changes in splicing regulation and *PICALM* risk SNPs rs3851179 and rs10792832 are associated with a specific reduction in the expression of isoform 1 but not isoform 2 [28,29]. Further *PICALM* SNPs have been associated with clinical features such as hippocampal atrophy, levels of Aβ and Tau in cerebrospinal fluid (CSF) and age of onset of disease [15,30–34]. PICALM is a ubiquitously expressed protein and is found throughout the brain including in neurons, microglia, oligodendrocytes and endothelial cells. Although cell type-specific functions of PICALM have begun to be investigated, there are still substantial gaps in our knowledge.

*PICALM* encodes the phosphatidylinositol-binding clathrin assembly protein (PICALM), which is key to CME and trafficking. PICALM is a highly abundant clathrin assembly protein responsible for sensing membrane curvature as well as controlling the size, maturation and completion of clathrin-coated vesicles [35,36]. The protein interacts with both clathrin and AP-2, bringing them together at the cell membrane, helping to initiate CME. PICALM has also been shown to regulate endocytic uptake of certain VAMP proteins that are key to autophagy, demonstrating that PICALM also has a role in maintaining functional autophagy [37–39].

### (a) Neurons

Unsurprisingly, as a crucial clathrin-interacting protein PICALM has been shown to have a role in synaptic vesicle recycling, regulation of synaptic vesicle size and trafficking of key synaptic proteins (figure 1, part 7). PICALM is expressed at both the pre- and post-synapse, with immunogold labelling localizing the protein to both synaptic and clathrin-coated vesicles as well as to endosomes in hippocampal neurons [40,41]. In line with this localization profile, reduction in PICALM expression in primary rat hippocamal neurons leads to a reduction in the number of synaptic vesicles and synaptic vesicle clusters, as well as causing enlargement of individual synaptic vesicles [42]. In addition to PICALM's role at the synapse, knockdown of PICALM in primary hippocampal neurons leads to fewer and shorter neurites giving rise to a less complex dendritic arbour [41].

Through PICALM's role in endocytic recycling it regulates the localization of VAMP2, an important synaptic protein required for the proper docking and release of synaptic vesicles (figure 1, part 7). In primary hippocampal neurons, the siRNA knockdown of PICALM causes the accumulation of VAMP2 at the cell surface, while overexpression in HEK293 cells reduces VAMP2 levels on the cell membrane [41,43,44]. Somewhat surprisingly, the authors of [43] report that this accumulation of VAMP2 at the neuronal membrane does not perturb neuronal excitability. However, this was measured using FM4-64 and so could be further explored using electrophysiological methods. PICALM has also been reported to alter the localization of VGlut in a *Drosophila* model in which expression of *lap,* the fly homologue of *PICALM*, rescued the accumulation of presynaptic VGlut observed upon Aβ42 expression [45]. Conversely, previous work in primary rodent hippocampal neurons did not see VGlut accumulation upon PICALM depletion, suggesting potential species disparities [43].

As well as PICALM causing changes to normal cellular functions that likely impact disease mechanisms, it has also been linked to both Aβ and Tau pathology. In an elegant study from the Lindquist laboratory, PICALM was found to be a modifier of Aβ toxicity. Using primary rat hippocampal neurons, PICALM expression rescued cells from Aβ-induced cell death in a dose-dependent manner [46]. Since this study, others have replicated PICALM's ability to modulate the toxic effects of Aβ [20,25,45]. PICALM has also been shown to associate with tau neurofibrillary tangles (NFT) in neurons of LOAD post-mortem brain samples, with more than 85% of NFT being colabelled with PICALM. In the same study PICALM was shown to immunoprecipitate with hyperphosphorylated tau from LOAD post-mortem brain samples [47]. Work following on from this has shown that Tg30xPicalm^+/−^ mice develop significantly more tau-positive neurofibrillary pathology throughout neurons of the brain compared to age-matched controls. PICALM has also previously been shown to modulate Tau levels in both *Drosophila* and zebrafish models [38].

Taken together, these data underline a key role for PICALM in synaptic homeostasis and suggest that changes in its expression levels impact normal neuronal function, potentially adding to disease pathogenesis as well as its participation in the Aβ and tau disease pathways.

### (b) Astrocytes

PICALM has been shown to have an important function in lipid homeostasis in astrocytes. Moulton and colleagues demonstrated when PICALM levels are reduced in rat primary astrocytes, but not neurons, there is decreased lipid droplet formation (figure 1, part 9) [48]. Previous work in HEK293 cells has also linked PICALM to lipid biology, showing that changes in its expression perturb cholesterol biosynthesis and lipoprotein uptake [49]. Moulton and colleagues show that when neuronal lipids were labelled in rodent primary cocultures in which astrocytes had normal or reduced PICALM expression, those with reduced PICALM levels showed a significant reduction in neuronal lipid uptake. This study demonstrates the importance of assessing phenotypes in a cell type-specific manner as well as investigating how changes in endocytic risk genes can alter cellular interactions.

PICALM is able to rescue APOE4-induced endocytic phenotypes in induced pluripotent stem cell (iPSC)-derived astrocytes [50]. Overexpression of PICALM was sufficient to rescue deficits in receptor-mediated endocytosis as measured using transferrin and EGF uptake assays. This overexpression was also enough to begin to increase early endosome antigen 1 (EEA1)-positive puncta size back towards normal size, which through APOE4 expression was reduced (figure 1, part 10). However, the authors noted that overexpression of PICALM in APOE3 astrocytes impaired endocytosis, demonstrating the careful balance in protein expression and endocytic activity required by these cells. This work shows an interesting interplay in two important LOAD risk genes and highlights the role of PICALM in orchestrating efficient endocytosis in human astrocytes.

### (c) Brain endothelia

PICALM can influence the clearance of A*β* from the brain through its function in the cells of the blood–brain barrier (bbb) (figure 1, §13). Zhao *et al.* [25] describe a 55–65% reduction in PICALM expression in cerebral microvessels of LOAD human post-mortem brain tissue, which correlates with diminished Aβ clearance and cognitive impairment. In *Picalm*^+/−^ mice there was a 61% reduction in Aβ42 efflux from the brain, whilst in AD-derived human brain endothelial cells the same group demonstrated a 35% reduction in PICALM expression and a 50% reduction in Aβ transcytosis, which could be recovered with adenoviral-mediated PICALM expression [25]. A recent study by Kisler *et al.* has corroborated these results, showing that *Picalm*^+/−^ 5xFAD mice have increased Aβ pathology and Aβ42 levels in the cortex and hippocampus [20]. Intriguingly when these mice were treated with artesunate, a compound able to increase PICALM expression, Aβ42 levels and pathology were reduced in the brain whilst Aβ42 blood serum levels were increased, suggesting accelerated clearance of Aβ over the bbb. Finally, specific depletion of endothelial Picalm abolished the effects of artesunate [20]. Both studies highlight the important role of PICALM in the role of transcytosis in brain endothelia and demonstrate the modulation of PICALM levels as a potential novel therapeutic strategy.

## 3. BIN1

GWAS studies have repeatedly identified *BIN1* as the second most significant susceptibility locus for LOAD after *APOE*, with multiple *BIN1* SNPs associating with cognitive decline and impaired memory [8–10,13,51–57]. The localization of these SNPs to a non-coding region suggests transcriptional dysregulation as a pathological culprit, supported by the presence of more than ten cell type-specific isoforms [58]. During CME, the BIN1 protein senses and induces membrane curvature and binds clathrin, AP-2 and dynamin [59]. BIN1 is expressed ubiquitously (BIN1iso9) but also has brain-specific isoforms [58]. All BIN1 isoforms contain the Bin/Amphiphysin/Rvs (BAR) domain, but the clathrin and AP-2-binding (CLAP) domain is found only in isoform 1 (BIN1iso1), expressed exclusively by neurons [60]. For further clarity regarding BIN1 isoform expression, we refer the reader to table 1.

**Table 1.** BIN1 isoforms and functions.

	**estimated molecular weight**^a^	**reported cell type-specific expression**^b^	**function**	**reported expression in AD**
**isoform 1**	∼80–95 kDa [1–4]	neurons [14,60–63]	CME via the CLAP domain [64] and exon 7 [65], possibly associated with tau pathology [58,62], presynaptic vesicle release dynamics [66]	decreased [14,60,61]
**isoform 2**	∼65–75 kDa	astrocytes [58,63]	possibly associated with tau pathology via exon 7 [58]	unknown
**isoform 3**	∼65–75 kDa	neurons [58,63]	possibly associated with tau pathology via exon 7 [58]	unknown
**isoform 4**	∼65 kDa	muscle [59]	non-CNS	n.a.
**isoform 5**	∼65 kDa	neurons, astrocytes [58,63]	unknown	unknown
**isoform 6**	∼65 kDa	microglia, neurons, astrocytes [58,67]	sensing and inducing membrane curvature via the bar domain [68], microglial pro-inflammatory response [67]	unknown
**isoform 7**	∼65 kDa	neurons, astrocytes [58,63]	unknown	unknown
**isoform 8**	∼65 kDa	muscle [59]	non-CNS	n.a.
**isoform 9**	∼55–60 kDa [14,61,66,67]	ubiquitous (includes neurons, astrocytes, microglia and oligodendrocytes) [14,61,66]	sensing and inducing membrane curvature via the BAR domain [68]	increased^c^ [14,66]
**isoform 10**	∼55–60 kDa	microglia, astrocytes [63,67]	sensing and inducing membrane curvature via the BAR domain [68], microglial pro-inflammatory response [67]	unknown
**isoform 12**	∼55–60 kDa	microglia, neurons, astrocytes	sensing and inducing membrane curvature via the BAR domain [68], microglial pro-inflammatory response [67]	unknown

^a^Citations are provided only for sizes of the BIN1 neuronal isoform 1 and the ubiquitous isoform 9, which have been confirmed in multiple publications, whereas estimations for the remaining isoforms are based on their exon content as their exact sizes have not yet been determined.

^b^Publication-to-publication disparities in reports of which combinations of cell types express which isoforms remain and should be noted.

^c^Increased expression in AD is typically reported for ‘short BIN1 isoforms’ and is generally attributed to the ubiquitous isoform 9 and glial isoforms, but isoform-specific expression changes have not yet been discerned, hence the other short isoforms have been labelled 'unknown' to avoid generalization.

### (a) Neurons

BIN1 has been shown to control EE formation in rat primary neurons, where BIN1 knockdown (KD) induced enlarged RAB5-positive endosomes and increased membrane-associated RAB5, indicative of increased endocytosis (figure 1, part 2). By contrast, overexpression of human BIN1iso1 resulted in reduced EE number [64]. The authors propose that this may be explained by the shared interaction of BIN1 and RAB5 with RIN3, a guanine nucleotide exchange factor (GEF), which is discussed in detail below.

A recent study reported conflicting results when expressing the human BIN1iso1 in *Drosophila* photoreceptor neurons, which normally lack a BIN1 homologue with the CLAP domain, observing neurodegeneration and accumulation of vesicles positive for early endosomal markers. The BIN1iso1-induced neurotoxicity was rescued by a dominant negative form of RAB5. Furthermore, knockout (KO) of *BIN1* in human iPSC-derived neurons decreased EE size, which was rescuable by re-expressing BIN1iso1 but not BIN1iso9 [69]. However, both studies still support a role for BIN1iso1 in control of EE size.

The role of BIN1 in CME may also have implications for Tau pathology spread. BIN1 silencing exacerbated propagation of phosphorylated Tau between synaptically connected rat primary neurons, whereas overexpression of human BIN1iso1, but not BIN1iso9, attenuated this effect. Tau was found to enter neurons via CME, with increased BIN1iso1 expression able to reduce Tau uptake [64]. The authors suggest this could be due to increased BIN1 gathering at sites of membrane curvature and over-recruiting dynamin, resulting in rigid and long budding necks, stalled vesicle formation, and consequently, less Tau internalization. However, one would expect that under conditions of reduced BIN1 expression there would be a failure to recruit dynamin, leading to a reduction in CME. Therefore, the mechanism by which this exacerbates Tau propagation in unclear. Furthermore, neurons of *Bin1* KO mice exhibited increased reserve pools of presynaptic vesicles and more vesicles docked at the presynaptic terminal with decreased release probability, alongside impaired learning and memory, indicating faulty synaptic vesicle recycling in mice lacking *Bin1* (figure 1, part 6) [66]. Altogether, these results suggest that BIN1 negatively regulates endocytic trafficking at the levels of the clathrin-coated vesicle and EE formation.

Furthermore, BIN1 has been demonstrated to interact with cytoplasmic Tau via the Src-homology-3 (SH3) domain [70]. Tau PS19 *Bin1* KO mice exhibited reduced survival and deficits in coordination and balance compared to PS19 controls. Antibody staining of the PS19 *Bin1* KO mice brains revealed region-specific differences in pathological Tau load, with a decrease in the hippocampus but an increase in the somatosensory cortex [71]. Interestingly, another mouse model overexpressing both human Tau and BIN1 also found decreased immunoreactivity against phosphorylated Tau in the hippocampus. However, these animals were protected against long-term cognitive deficits seen in mice expressing Tau only. The authors then characterized BIN1's interaction with Tau by immunostaining and immunoblotting of primary rat neuronal cultures. They demonstrated that when phosphorylated, BIN1 takes up an open conformation favouring Tau binding, and also found an increased ratio of phosphorylated to un-phosphorylated BIN1 in extracts from human AD brains [72].

In contrast to the clear link between BIN1 and Tau in AD, BIN1 involvement in Aβ pathology is less well defined. PET imaging of subjects carrying the lead *BIN1* risk SNP have produced conflicting results regarding whether this status is associated with greater Aβ load [13,56]. Moreover, no effect was seen in *Bin1* KO mice or in a human neuroblastoma cell line following *BIN1* silencing or overexpression [61,73]. However, concomitant depletion of BIN1 and CD2AP from mouse primary neurons increased intracellular Aβ42 accumulation, while BIN1iso1 overexpression in N2A cells decreased Aβ42 levels but only when co-expressed with RIN3, suggesting that the role of BIN1 in Aβ pathology may be dependent on other proteins [74,75].

### (b) Microglia

Microglia express BIN1iso6, BIN1iso10, BIN1iso12 alongside BIN1iso9, which is elevated in human AD brain homogenates [14,58,61,62,76]. However, bulk tissue measurements are not enough to account for this increase to microglia. Microglial BIN1 isoforms lack the CLAP domain, but retain the BAR domain, suggesting involvement in membrane curvature dynamics [60].

Recent evidence has underlined the importance of studying the role of BIN1 in microglia as fine mapping of AD variants revealed that several *BIN1* SNPs reside in microglia-specific regulatory regions upstream of the *BIN1* transcription start site [77–82]. This includes a key microglial enhancer encompassing the lead *BIN1* risk SNP characterized by Nott and colleagues [77]. Deletion of this region in iPSC resulted in a drastic reduction in BIN1 expression specifically in iPSC-derived microglia, but not in astrocytes or neurons [77].

The function of microglial BIN1 has not been well studied. To investigate BIN1 involvement in the microglial inflammatory response, Sudwarts and colleagues performed transcriptomic profiling of BIN1-depleted primary mouse microglia and microglia isolated from *Bin1* KO mice [67]. BIN1 loss led to widespread downregulation of proinflammatory genes, inclusive of genes related to the disease-associated microglia phenotype, notably including Apoe (figure 1, part 12). Furthermore, BIN1 loss attenuated the effects of lipopolysaccharide (LPS) treatment on proinflammatory gene expression in *Bin1* KO microglia and dampened their ability to respond to LPS. These microglia retained ramified morphology and failed to exhibit increased phagocytosis normally seen in response to LPS [67]. Taken together, these findings indicate that under homeostatic conditions, BIN1 positively regulates several proinflammatory genes, in turn mediating microglial inflammatory responses, which are involved in AD [83].

### (c) Oligodendrocytes

BIN1iso9 is highly expressed by oligodendrocytes, and *BIN1* risk variant eQTLs have been recently located to oligodendrocyte-specific regulatory regions [60,82]. The role of BIN1 in oligodendrocytes in uncharacterized, but De Rossi and colleagues reported upregulation of BIN1 in postnatal rat brains at periods of peak myelination, and during the oligodendrocyte maturation stage [60].

### (d) Astrocytes

In addition to short glial BIN1 isoforms, BIN1iso2 and BIN1iso3—which contain exon 7**—**have been detected in astrocytes [58,63]. Exon 7 modulates BIN1's interaction with dynamin during CME [60]. Proteomics revealed that reduction of exon 7-derived peptides correlated with worsening of AD-related cognitive decline and increased Tau and amyloid burden. As BIN1iso1 also contains exon 7, neuron loss is a possible explanation, but Taga and colleagues argue in support of an astrocyte-specific effect, based on observations of selective loss of astrocytes expressing exon 7 containing BIN1 in human AD brains versus controls, without differences between groups in the total number of astrocytes or the number of neurons expressing BIN1 [58]. However, another study failed entirely to detect BIN1 in astrocytes [60].

## 4. CD2AP

Despite some instances of deviation across differing populations (reviewed in [84]), the rs9349407 locus of *CD2AP* has been shown to have a genome-wide significant association with LOAD risk [8,10,84,85]. CD2AP (CD2-associated protein) encodes an adaptor protein consisting of three consecutive SH3 motifs, a proline-rich region and a carboxy terminus that acts as an actin-binding domain [86]. This actin-binding domain highlights the role of CD2AP in the regulation of the cytoskeleton and thus endocytosis, potentially as an adapter between the actin cytoskeleton and other cell membrane proteins [87,88].

In neurons, CD2AP displays a conserved role in endocytosis and synaptic transmission demonstrated in humans, mice and in *Drosophila*. Using the CD2AP fly orthologue *cindr*, Ojelade and colleagues found that *cindr* was associated with synaptic proteins and caused impairments in synaptic endocytosis through a conserved role in the regulation of the ubiquitin–proteasome system in synaptic proteostasis [89]. Through shRNA knockdown experiments using *in vitro* N2a neuroblastoma cultures transfected with human APP, it was shown that reduced CD2AP expression results in a decrease in cell membrane APP, which would suggest less APP being processed in the endosomes and thus decreasing Aβ release [90]. These findings could not be recapitulated *in vivo* using Cd2ap haploinsufficiency PS1APP mice up to 7 months of age, however it should be noted that this is a relatively young time point thus warranting further study in aged mice.

CD2AP has been linked to axonal outgrowth through its colocalization with TrkA and Rab5-labelled EEs, which suggests a role of CD2AP in long-range nerve growth factor (NGF) signalling via TrkA endocytosis to activate the AKT pathway, resulting in the regulation of collateral sprouting [91].

In primary neuronal cultures, CD2AP has been shown to work in tandem with the well-known endocytic risk gene *BIN1* in the dendrites and axons. BIN1 regulates BACE1 recycling in the axons, and when depleted, results in Aβ accumulation in the axons. CD2AP plays a similar role in the dendrites so that when CD2AP levels are reduced, APP is trapped at the limiting membrane of EE and fails to be sorted for degradation (figure 1, part 8). This study demonstrates that these two key AD genetic risk factors work to differentially polarize Aβ generation during endocytosis [74].

CD2AP has also been shown to play a conditional role in endosomal enlargement in non-neuronal cells as a Rab4 effector. Using Chinese hamster ovary (CHO) cells, CD2AP was shown to specifically interact with Rab4-GTP and c-Cbl, and co-expression of CD2AP with both resulted in enlarged EEs morphology suggesting a role in sorting from EEs to LEs [92].

## 5. RIN3

Another recently identified candidate AD risk gene that is associated with vesicle trafficking to EEs is *RIN3* (Ras and Rab interactor 3) [79]. When coupled with other AD endocytic risk genes, particularly *BIN1*, it has been implicated in both Aβ and tau pathology.

Rab GTPases play critical roles in endocytosis, but this role is governed by guanine nucleotide binding modulated by GEFs. The role of RIN3 in the recruitment of BIN1 to the EE was first highlighted by Kajiho and colleagues in 2003 [93], where it was shown that RIN3 is in fact a key partner to BIN1 in early endocytosis. This study demonstrated that the proline-rich N-terminus of RIN3 directly associates with the BIN1 SH3 domain [93]. They have since further characterized RIN3 function as a GEF to stimulate and stabilize Rab5 and its subfamily proteins, particularly Rab31 [94]. The RIN3/BIN1 interaction has been further elucidated *in vitro* showing RIN3 to selectively recruit the neuronal CLAP domain-containing BIN1iso1 isoform to Rab5-positive endosomes. Neuro 2A cells overexpressing both BIN1iso1 and RIN3 displayed markedly reduced A*β* generation compared to overexpression of BIN1iso1 alone, which altogether suggests that the neuronal-specific BIN1 effects on APP endocytosis, both trafficking and processing, are RIN3-dependent [75].

Overall, characterization of exactly how an increase in RIN3 expression plays a role in Rab5 activity and consequently, AD pathology is still in its infancy. However, early endosomal enlargement alongside consistently upregulated RIN3 mRNA levels has been demonstrated in basal forebrain cholinergic neurons of APP/PS1 mice. Shen *et al.* have demonstrated a role for RIN3 as a regulator of axonal trafficking by acting as a scaffold tethering Rab5 EEs to BIN1 and CD2AP. They showed that increasing RIN3 expression resulted in an increase in both *β*CTFs and pTau levels in PC12 cells and posited that the RIN3/CD2AP interaction results in Aβ pathology, while the RIN3/BIN1 interaction linked to the tau pathology [95].

## 6. SORL1

*Sortilin-related receptor 1* (*SORL1*) is a notable AD gene due to its association with both early-onset familial AD (through inherited SORL1 haploinsufficiency) and late-onset AD (LOAD; when *SORL1* was identified as a risk gene in multiple GWAS) [8,11,96–99]. The full-length transcript of *SORL1* encodes a 250 kDa, membrane-bound protein that functions as a key endocytic sorting and trafficking receptor primarily localized in the endosomal and Golgi compartments [100]. The receptor has also been shown to bind lipoproteins, mediating their uptake via endocytic pathways [101]. SORL1 has been demonstrated to be important in the regulation of APP sorting between either the retrome–RE pathway or the endosome–lysosome pathway, the latter resulting in APP cleavage and thus, Aβ generation [102]. Mutations in the *SORL1* gene can therefore alter the sorting of the receptor ligands/cargo, ultimately resulting in increased Aβ production [103].

In LOAD, SORL1 expression is downregulated and has been shown to lead to early endosomal enlargement in human iPSC-derived neurons [104–107]. It was shown that SORL1 depletion results in a decrease of APP localization within the trans-Golgi network (reduced recycling) and the retention of APP in the EEs (figure 1, part 4) [108]. The importance of SORL1 in endocytic sorting is further supported with reduced trafficking of additional SORL1 cargo, the glutamate receptor out of the EE to the LE and lysosome [109]. By contrast, overexpression of SORL1 displayed enhanced endosomal recycling through redirection of APP to the Golgi-network, thus blocking APP processing and reducing Aβ peptide levels in neuroblastoma, mouse and human induced pluripotent stem cell (hiPSC)-derived neuronal models [102,104,109,110].

In addition to APP, SORL1 has additional cargo that when misdirected can have cell-specific adverse effects, including dysregulated synaptic transmission. SORL1 has been associated with glutamate receptor recycling to the cell surface in iPSC-derived neurons and in the trans-entorhinal cortex in mice by interacting with VPS26b, a retromer subunit [109,111].

In humans, *SORL1* is most highly expressed in microglia [105]. Using BV2 microglia and neuroglioma cells, Chen and colleagues identified SORL1 as a binding partner to progranulin [112]. SORL1 ablation has been shown to rescue neurodegeneration-associated decreases in progranulin (PGRN) binding, suggesting a role for SORL1 in the regulation of progranulin endocytosis and clearance. Progranulin is a diverse growth factor associated with the anti-inflammatory control of microglial functions, including specialized forms of endocytosis such as phagocytosis and synaptic pruning [113]. In addition to the cell type-specific roles, the cell-to-cell interaction effects of endocytic risk genes are interesting, as knocking out SORL1 in neurons reduces binding and thus endocytosis of microglia-secreted progranulin. This has also been demonstrated *in vivo* using heterozygous knockout *Pgrn* mice models of frontotemporal lobar degeneration, where SORL1 ablation rescued the disease-causing PGRN decrease through reduced endocytosis [114]. However, the induction of the characteristic early endosomal enlargement through the loss of SORL1 was shown to be specific to iPSC-derived neurons but not recapitulated in iPSC-derived microglia [108]. This further highlights the importance of understanding cell type-specific effects of endocytic dysfunction.

## 7. APOE

**Figure 2.**
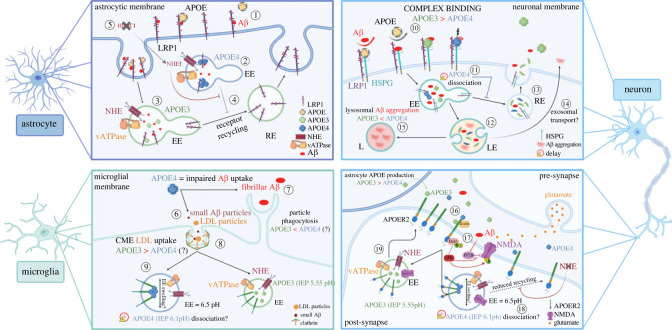
Cell type-specific roles of APOE in the endocytic pathway. Although APOE is not a bona fide endocytic protein, LOAD-associated changes cause endocytic phenotypes. (1) In astrocytes, APOE and A*β* bind to the metabolic receptor LRP1 (low density lipoprotein receptor-related protein 1). Particles are taken into the cell where they dissociate from their receptors within the EE. APOE4 (2) compared to APOE3 (3) delays dissociation by clustering around receptor sites and reducing receptor recycling (4). This effect is counteracted by HDAC (histone deacetylase) inhibition (5). In microglia the APOE4 isoform has been shown to impair uptake of small Aβ particles (6) via CME and fibrillar Aβ (7) via phagocytosis, as well as causing a reduction in LDL uptake compared to APOE3 (8). Delayed APOE4 receptor dissociation is additionally thought to play a role in reduced particle uptake and swelling of the early endosome (EE) (9). In neurons, APOE competes for binding with A*β* at LRP1–HSPG (heparan sulfate proteoglycan 2) receptor sites and more stable APOE3/A*β* complexes are taken up more readily then less stable APOE4/A*β* (10) complexes at these sites. Endocytosed APOE dissociates from its receptors within the EE, with APOE4 showing delayed dissociation (11). After dissociation, particles are transported to the late endosome (LE) (12) or are recycled back to the extracellular space by the recycling endosome (13). Some particles including A*β* aggregates reach the extracellular space via exosomes (14). From the LE, particles are mainly transported to the lysosome for degradation, where a low pH is believed to contribute to augmented Aβ-aggregation in APOE4 neurons (15). At the synapse, APOER2 provides dual binding to APOE and Reelin (16). Reelin activates SFK upon binding, leading to GluN2 phosphorylation enhancing NMDA activity. A*β* simultaneously suppresses NMDA activation (17). In APOE4 neurons reduced receptor recycling is linked to the low IEP differential between the EE (6.5pH) and the APOE4 isoform (6.1pH), this has been counteracted by NHE inhibition which increases the IEP differential restoring receptor recycling (18). Accurate receptor recycling, as seen in APOE3 neurons (18), is therefore essential for glutamatergic neurotransmission (19).

Apolipoprotein E (APOE) is the primary lipid-transporter protein in the brain and APOE4 represents the largest genetic risk factor for developing LOAD. APOE occurs in three isoforms; *APOE2*, *APOE3* and *APOE4*, characterized by differing isoelectric points (IEP) resulting from single nucleotide polymorphisms at amino acid positions 112 and 158 [115–118]. The different IEPs further lead to differential binding preferences, with APOE3 and APOE2 favourably binding high-density lipoproteins (HDL), while APOE4 shows higher association to very low-density lipoproteins (VLDL) and low-density lipoproteins (LDL) [119,120]. Within the brain APOE is predominantly expressed by astrocytes, followed by microglia, and neurons with low expression. Among the human isoforms, APOE3 is the most prominent with a neutral risk-association, whereas the APOE2 isoform results in a 50% decrease of LOAD risk. Homozygosity of *APOE4* gives a 12-fold increase in risk of developing LOAD, whereas heterozygous carriers have a threefold increased risk [115]. This universal risk recognition has prompted research to predominantly focus on APOE's contribution in disease progression by looking at comparative phenotypes among isoforms. Such research has provided important insights into isoform-dependent phenotypes but underlined our lack in understanding of the mechanisms of APOE in a healthy context. However, more recent research has started to investigate such mechanistic differences and, although not traditionally considered a bona fide endocytic risk gene, found a role of APOE in the endosomal–lysosomal pathway (ELP), specifically as a mediator in receptor recycling and a contributor in early endosomal enlargement [121]. The roles of APOE in endocytosis in different cell types are summarized in figure 2.

### (a) Astrocytes

Astrocytes are the main producers of APOE, making them essential in the maintenance of lipid-homeostasis. The expression of APOE from astrocytes is further dependent on the APOE-isoforms. iPSC models identified lower APOE generation in *APOE4* compared to *APOE3* iPSC-derived astrocytes, an effect that was accompanied by a decrease in the number of EEA1 puncta in *APOE4* astrocytes and an increase in the number of EEA1-puncta in *APOE4* iPSC*-*derived neurons, suggesting a role of APOE within the ELP [50,122]. Interestingly, *APOE4* iPSC-derived astrocytes showed impaired early endocytosis, with a reduction in EEA1 protein levels and CME compared to *APOE3* astrocytes [50]. No impact was seen in REs, and only a marginal decrease of LEs and lysosomes was observed in *APOE4* astrocytes, strongly suggestive of an early role of APOE within the early endocytic pathway [122]. However, APOE4-induced endocytic impairments were abolished through *APOE4* knockout lines as well as PICALM overexpression, as discussed above. The reduction of RAB5- and EEA1 - positive EEs observed in *APOE4* iPSC-derived astrocytes was contradictory to findings in *APOE4* iPSC-neurons and *APOE4* yeast homologues demonstrating cell type-specific effects [50,122].

Aside from being the main APOE producers in the brain, astrocytes alongside microglia are crucial for the uptake and endocytosis of substrates from the extracellular space including Aβ, tau and α-synuclein [123]. Aβ uptake/clearance in astrocytes is facilitated via lipid-associated endocytic receptors such as LDLR or LRP1 and has been shown to occur via APOE isoform-dependent complex binding, with -POE2/Aβ and APOE3/Aβ complexes resulting in faster clearance than APOE4/Aβ complexes in the mouse brain [124–126]. Indeed, reduced LRP1 expression and Aβ uptake in astrocytes was linked to receptor entrapment in the EEs due to late APOE4 receptor dissociation in the EE, an effect that was counteracted through inhibition of nuclear histone deacetylation 4 (figure 2, parts 2–4) [127]. A reduction of complex-dependent Aβ uptake was further detected in *APOE4* iPSC-derived astrocytes but this was not affected by LRP1 blockage, opposing Prasad & Rao's previous findings [127]. Lin and colleagues likewise detected decreased Aβ42 uptake in *APOE4* iPSC-derived astrocytes, accompanied by a reduction in lysosomal Aβ42 [122]. Again, these findings show conflicting results to those observed in mouse primary neurons, where NHE6 inhibition led to restored Apoer2 recycling and restored NMDA activity [117]. Such continuous incongruity in neuronal and astrocytic findings could indicate cell type-specific endocytic dysfunction and consequently more research is needed to unfold these mechanisms and their intercellular complementation.

### (b) Microglia

Microglia are consistently reported to be one of the largest producers of APOE within the brain [129,130]. The influence of APOE on microglia is commonly reported in the context of phagocytosis, a specialized form of endocytosis that microglia use to carry out homeostatic functions in clearing debris [24]. Unlike neurons and astrocytes, limited studies specifically investigate the mechanistic role of APOE in microglial endocytic dysfunction.

Transcriptionally, increased expression of *APOE* is observed in microglia that have altered expression profiles related to neurodegenerative disease changes and ageing. Such microglia have been given various names, including disease-associated microglia (DAM), microglial neurodegenerative phenotype (MGnD) or activated response microglia (ARMs), which are all linked to an upregulated phagocytic phenotype [131,132]. Hypotheses that APOE is an opsonizing agent in this context, inducing phagocytosis in microglia, have been explored. Higher levels of APOE were bound to dead neurons compared to healthy neurons, and their uptake by primary mouse microglia was increased in the presence of APOE, implying opsonization [133]. In TREM2-KO primary microglia, for which APOE is a ligand, phagocytosis was reduced, confirming that APOE and TREM2 collaborate to facilitate phagocytosis of dead neurons [133].

In mice, injection of Aβ conjugated to APOE4 lipoproteins into the cortex caused impairment in Aβ uptake by microglia at 24 h, compared to APOE3, and APOE expression was upregulated in phagocytic microglia [134]. In primary microglia, this result was replicated at 1 h using oligomeric Aβ42 [134]. Muth *et al.* showed, using *in vitro* murine microglia, that APOE4 increases apoptotic neuron phagocytosis, but reduces oligomeric Aβ phagocytosis after 30 min, compared to APOE3 (figure 2, parts 6–7) [135]. These confirmed results from Lin *et al.* in an iPSC-derived microglia model [122]. The size of Aβ particle is not reported in several of these studies, therefore limited conclusions can be drawn. Due to size discrimination, fibrillar Aβ would be taken up by phagocytosis while smaller oligomeric forms would be internalized through CME [24]. Moreover, several of these conclusions were drawn from single timepoint experiments and were based on total intracellular levels of cargo, which may lead to misinterpreted results. Jiang *et al.* showed that total Aβ uptake is not affected by APOE isoform in a murine *in vitro* model, but Aβ degradation through the endocytic pathway is hindered by APOE4 [136]. Contradicting results from Jiang *et al.* [136] and Fitz *et al.* [134] highlight the importance of experimental design in elucidating true endocytic function in microglia, which has been reviewed elsewhere [24].

Machlovi *et al.* [137] performed detailed pHrodo-based endocytosis assays over several timepoints, in which myelin, apoptotic cells, zymosan particles and latex beads are all phagocytosed to a higher extent in APOE4 primary mouse microglia compared to APOE3. In *APOE4* iPSC-derived microglia, however, a reduction in low-density lipoprotein uptake by CME was observed at 1 h (figure 2, §8) [138]. These data highlight the cargo- or species-dependent nature of endocytic activity in microglia, and that APOE may have differential effects on distinct endocytic pathways.

Regarding specific mechanistic alterations, in APOE4 primary mouse microglia, increased RAB5 and RAB7 staining is observed, along with increased lysosomal mass and decreased pH [137]. Knock-down of Abca1 depleted the ability of primary microglia to lipidate APOE and reduced the degradation of Aβ [136]. Suggesting that increased glial activation and Aβ-plaque reduction is observed following sodium/hydrogen exchanger 6 (NHE6) depletion, demonstrating the IEP hypothesis in microglia, requires further investigation [117].

### (c) Neurons

Despite low neuronal APOE secretion compared to astrocytes and microglia, cell non-autonomous APOE signalling is highly important for neuronal function. Indeed, APOE receptor expression is essential for synaptic cholesterol transport and dependent on accurate receptor recycling within the ELP [116]. The EE's slightly acidic environment [6.4 pH], regulated via the vATPase-dependent proton pump and the NHE6 proton leak, is essential for receptor–ligand dissociation and consequent sorting into recycling vesicles [117,127,139]. Recent evidence linked APOE4 to delayed receptor dissociation, which is possibly caused through its tendency to form a molten globule when exposed to acidic environments [140]. Additionally, APOE4 retention contributes to EE enlargement and accumulation at pre-symptomatic disease stages [16,122]. Cataldo *et al*. [16] found enlarged EEs in neurons of post-mortem samples of pre-clinical Alzheimer's patients. This EE enlargement was accelerated in patients holding one or two copies of the *APOE4* allele [16]. Likewise, Lin *et al*. [122] detected higher EEA1 levels in *APOE4* iPSC-derived neurons compared to *APOE3*. In fact, the pH of the EE is almost equivalent to the IEP of APOE4, leading to an IEP surface charge reduction resulting in decreased solubility that slows APOE4 receptor dissociation and diminishes receptor recycling (figure 2, part 11) [141]. Loss of surface receptors can subsequently impact cellular communication and clearance mechanisms. The Apoer2 receptor, for example, has a dual binding function with Reelin and APOE. Upon binding to Apoer2, Reelin activates Src family non-receptor tyrosine kinases (SFKs), leading to the phosphorylation of GluN2 subunits and enhancing NMDA activity in combination with long-term potentiation (LTP) [142]. Reduced APOER2 receptor recycling accordingly impacts reelin-mediated NMDA activity and enhances Aβ-mediated NMDA-suppression, which is counteracted by increasing the IEP differential between APOE4 and EE through NHE6 inhibition [141,143]. Incubation of primary cortical neurons from APOE humanized mice with recombinant human APOE4 significantly increased intracellular retention of APOE4 lipoproteins and reduced cell-surface levels of APOER2, resulting in the abolition of Reelin-induced LTP while also enhancing Aβ-induced suppression of LTP [142]. This was confirmed by Pohlkamp *et al*. [117], who found that NHE6 ablation *in vivo* enhanced Apoer2 recycling to the synapse in APOE4 targeted replacement mice (figure 2, §§16–19). Furthermore, the authors found Aβ-plaque reduction in these animals and in humanized APP mice, suggesting enhanced uptake following NHE6 depletion regardless of the APOE4 phenotype [117]. The above findings imply a possible mechanism by which APOE could interrupt synaptic neurotransmission and speed up synaptic dysfunction in disease.

One of the major receptors responsible for Aβ uptake is the metabolic APOE receptor LRP1 [116]. APOE and Aβ compete for binding at LRP1-HSPG receptor sites, with HSPG showing a preference for Aβ [144,145]. Moreover, APOE can aid neuronal Aβ uptake by forming APOE/Aβ-complexes whereby APOE3 builds more stable complexes than APOE4 [146,147]. Although LRP1 deletion has been linked to augmented Aβ half-life and enhanced Aβ-amalgamation, a recent study found that LRP1-knockout APP/PS mice with humanized *APOE4* showed reversal of Aβ-deposition and increase in soluble APOE4 [148,149]. In the absence of LRP1 Aβ-seeding might be averted through APOE's association with the HSPG receptor preventing Aβ binding from causing less endocytosis of APOE and increased Aβ degradation [150]. Interestingly, Van Gool *et al*. [151] found that impaired LRP1 function *in vivo* modified APP processing from β-secretase to α-secretase cleavage, which led to diminished Aβ and Aβ-plaque load. Furthermore, Lin *et al*. [122] found a 20% increase of Aβ42 in APOE4 iPSC lines compared to APOE3 lines. In this sense, reduced endocytosed LRP1 receptors seem to have a strong effect on Aβ production, which could potentially outweigh the simultaneous reduction of LRP1-Aβ clearance mechanisms.

## 8. Conclusion

The importance of understanding the mechanisms that lead to the development of LOAD cannot be understated and will help overcome the current bottleneck to producing effective treatments. As discussed throughout this review, both genetics and pathology converge on the importance of endocytic dysfunction in LOAD and as more genetic studies continue it is likely that further genes will be identified within this pathway. Endocytic and endosomal dysfunction are seen across the neurodegenerative spectrum, demonstrating the essential role of maintaining effective cellular recycling and trafficking in the brain, particularly in old age.

Across the LOAD endocytic risk genes discussed throughout this review, it is apparent that there is convergence of potential pathogenic mechanisms. In all cases changes in expression of these endocytic genes have been shown to modulate Aβ and/or tau aggregation, clearance and toxicity (see electronic supplementary material, table S1 for a summary). Another commonality is that expression changes in these genes causes alterations to the size of the EE. Enlarged EEs are a pathological hallmark of the LOAD brain. Although it remains unclear what this means more broadly in terms of EE function, previous work has demonstrated that poor trafficking due to reduced retromer-dependent endosomal recycling can lead to this endosomal enlargement, as can dysfunction of APP processing [108,152]. It is likely that the above-discussed genetic changes alter endosomal size through similar mechanisms, with evidence already for *CD2AP*, *SORL1* and *BIN1* altering APP processing [74,75,90,102,108]. In addition, these changes in endocytic and endosomal function have wider cellular impacts leading to cellular dysfunction, which are often overlooked but important to understand in a disease context. For example, altered neuronal firing and synaptic organization, astrocytic lipid homeostasis and microglial inflammatory profiles are all impacted by alterations in the endocytic genes discussed above [40–42,49,66,67,109]. Previous work has often concentrated on the neuronal function of endocytic risk genes, but as is ever apparent other cell types are also important in LOAD and therefore warrant further investigation moving forward. It is likely that endocytic and endosomal changes also impair cellular interactions directly through changes to membrane receptor compositions and/or cellular signalling cascades. Impairment of endocytic activity in one cell type, such as microglia, may be able to begin to drive pathogenic phenotypes in other cell types in an indirect manner too. For example, impaired microglial clearance of pathogenic Aβ leads to its build-up, which in turn is able to impact neuronal firing properties [153,154]. Such cellular interactions require examination through more complex coculture systems in future work.

SNPs in these LOAD endocytic risk genes are known to have small effect size individually. However, when multiple such genetic risk factors are inherited together converging on the same pathway it is likely that a threshold for the development of LOAD is reached in terms of endocytic/endosomal impairment. As discussed in this review, some evidence of how such risk genes may interact to produce a culminative impact already exists, e.g. *BIN1/CD2AP*, *APOE/PICALM*, *BIN1/RIN3*, but this is an area that certainly requires further understanding [50,74,75,95].

Throughout this review, we highlight how endocytic and endosomal dysfunction impacts different cell types; however, it is evident that further work is required to gain a broader and more nuanced insight into how this impacts cell type-specific function. Importantly, endocytic/endosomal dysfunction appears to be an early mechanism of disease, likely in part to be independent of other hallmark pathologies such as Aβ and tau aggregation, and so presents itself as an attractive target for much needed disease modifying therapies.

## Data Availability

The data are provided in electronic supplementary material [155].
